# The accuracy of pulse oximetry in emergency department patients with severe sepsis and septic shock: a retrospective cohort study

**DOI:** 10.1186/1471-227X-10-9

**Published:** 2010-05-05

**Authors:** Ben J Wilson, Hamish J Cowan, Jason A Lord, Dan J Zuege, David A Zygun

**Affiliations:** 1 Department of Medicine, University of Calgary, (1403 - 29th Street NW), Calgary, (T2N 2T9), Canada; 2 Faculty of Medicine, University of Calgary, (1403 - 29th Street NW), Calgary, (T2N 2T9), Canada; 3 Department of Critical Care Medicine, University of Calgary, (1403 - 29th Street NW), Calgary, (T2N 2T9), Canada; 4 Division of Emergency Medicine, University of Calgary, (1403 - 29th Street NW), Calgary, (T2N 2T9), Canada; 5 Department of Clinical Neurosciences, University of Calgary, (1403 - 29th Street NW), Calgary, (T2N 2T9), Canada; 6 Department of Community Health Sciences, University of Calgary, (1403 - 29th Street NW), Calgary, (T2N 2T9), Canada

## Abstract

**Background:**

Pulse oximetry is routinely used to continuously and noninvasively monitor arterial oxygen saturation (SaO_2_) in critically ill patients. Although pulse oximeter oxygen saturation (SpO_2_) has been studied in several patient populations, including the critically ill, its accuracy has never been studied in emergency department (ED) patients with severe sepsis and septic shock. Sepsis results in characteristic microcirculatory derangements that could theoretically affect pulse oximeter accuracy. The purposes of the present study were twofold: 1) to determine the accuracy of pulse oximetry relative to SaO2 obtained from ABG in ED patients with severe sepsis and septic shock, and 2) to assess the impact of specific physiologic factors on this accuracy.

**Methods:**

This analysis consisted of a retrospective cohort of 88 consecutive ED patients with severe sepsis who had a simultaneous arterial blood gas and an SpO_2 _value recorded. Adult ICU patients that were admitted from any Calgary Health Region adult ED with a pre-specified, sepsis-related admission diagnosis between October 1, 2005 and September 30, 2006, were identified. Accuracy (SpO_2 _- SaO_2_) was analyzed by the method of Bland and Altman. The effects of hypoxemia, acidosis, hyperlactatemia, anemia, and the use of vasoactive drugs on bias were determined.

**Results:**

The cohort consisted of 88 subjects, with a mean age of 57 years (19 - 89). The mean difference (SpO_2 _- SaO_2_) was 2.75% and the standard deviation of the differences was 3.1%. Subgroup analysis demonstrated that hypoxemia (SaO_2 _< 90) significantly affected pulse oximeter accuracy. The mean difference was 4.9% in hypoxemic patients and 1.89% in non-hypoxemic patients (p < 0.004). In 50% (11/22) of cases in which SpO_2 _was in the 90-93% range the SaO2 was <90%. Though pulse oximeter accuracy was not affected by acidoisis, hyperlactatementa, anemia or vasoactive drugs, these factors worsened precision.

**Conclusions:**

Pulse oximetry overestimates ABG-determined SaO_2 _by a mean of 2.75% in emergency department patients with severe sepsis and septic shock. This overestimation is exacerbated by the presence of hypoxemia. When SaO_2 _needs to be determined with a high degree of accuracy arterial blood gases are recommended.

## Background

Pulse oximetry is a routine part of the monitoring and management of critically ill patients [1]. Studies have proposed that specific pulse oximter oxygen saturations (SpO_2_) be targeted to decrease the likelihood of hypoxemia [1-4], to titrate fractional inspired oxygen [5], and to wean mechanical ventilation [6].

The accuracy of pulse oximetry to estimate arterial oxygen saturation (SaO_2) _in critically ill patients has yielded mixed results. Both the degree of inaccuracy, or bias, and its direction has been inconsistent [1-3,5,7-9]. In addition, while certain studies of critically ill patients have demonstrated that hypoxemia [1], anemia [10], requirement for vasoactive drugs [7], and acidosis [8] influence the accuracy of pulse oximetry, others have not [2,6]. Data on the effects of other physiologic derangements, such as hyperlactatemia and bacteremia, are absent.

Pulse oximeters utilize the pulsatile nature of arterial blood flow to distinguish it from venous flow and estimate oxygen saturation in arterial blood [11]. Processes that increase venous blood flow or alter pulsatility can interfere with the ability of pulse oximeters to estimate arterial oxygen saturation. Hemodynamic derangements in septic patients, such as arteriovenous shunting, cutaneous arteriolar dilation and decreased vascular resistance [9,12] can alter pulsatility and venous blood flow and therefore theoretically affect pulse oximeter accuracy. When reproduced in healthy volunteers [13], cutaneous vasodilation has been shown to interfere with the pulse oximetry signal and significantly decrease its accuracy. This has also been demonstrated in animal models of severe sepsis [14,15]. The two existing studies examining the performance of pulse oximetry in humans with septic shock [7,9] were small, consisting of a combined 17 patients, and were undertaken in the intensive care unit (ICU), later in the course of disease. As the pathophysiology of sepsis evolves over time, with its distinct temporal changes to hemodynamic [16] and inflammatory [17] variables, there is an important paucity of data regarding pulse oximeter accuracy early in the course of severe sepsis. As tissue hypoxia drives sepsis-induced organ failure and death [18,19], reliable detection and correction is of these derangements is critical in patients with severe sepsis. Pulse oximeter performance has never been studied in ED patients with severe sepsis and septic shock.

The present analysis is part of a research program aimed at determining factors associated with the development of acute lung injury in patients with severe sepsis and septic shock. This study aims to determine the accuracy of pulse oximetry in emergency department patients with these disease states and to determine the effects of specified physiologic derangements on this relationship.

## Methods

This study protocol was approved by the University of Calgary Conjoint Health Research Ethics Board (Ethics ID# 21548). The study sample involved a retrospective cohort that included consecutive adult patients admitted to these three ICUs directly from the ED, with a sepsis-related Intensive Care National Audit & Research Centre (ICNARC) diagnosis between October 1, 2005 and September 30, 2006. Patients were identified from a local longitudinal ICU database known as TRACER (Microsoft Access, Microsoft Corporation, Seattle, WA, USA). Inclusion criteria were age ≥ 18 years, admission directly from the ED, and patients must have met standard conventional definitions for severe sepsis or septic shock [20]. Specifically, all patients had evidence of infection, two or more systemic inflammatory response syndrome criteria (temperature: either > 38°C or < 36°C; heart rate > 90; respiratory rate > 20 breaths/min or PaCO2 < 32 mmHg; white blood cell count: > 12000 cells/mm^3^, < 4000 cells/mm^3^, or > 10% bands), and either organ dysfunction, as defined by Ferreira [21], or systolic blood pressure < 90 mmHg. Exclusion criteria included signs of left atrial hypertension, congestive heart failure, chronic lung disease, and etiologies of non-septic acute lung injury (pancreatitis, aspiration pneumonia, or traumatic pulmonary contusion).

Charts were reviewed for ED values of pulse oximetry, which is standard of care in our regional EDs, and results of the corresponding initial arterial blood gas. The pulse oximetry value recorded at the time of the ABG was used. It was standard practice for respiratory therapists to record the SpO_2 _at the time that the ABG was drawn. Data extracted included: SaO_2_, SpO_2_, serum lactate, hemoglobin from the first complete blood count drawn in the ED, ED blood culture result, and whether a vasoactive agent was administered in the ED. Only the values from the first ABG were used. Incomplete data sets, including those arising from pulse oximeter signal failure, were excluded.

Pulse oximetry readings were recorded using a Nellcor pulse oximeter (N20, N65, N75, N85, NPB40, or NPB 40 MAX, Hayward, California) using DS 100A finger probes were attached to a finger and were not necessarily on the arm from which the arterial blood was sampled. Arterial blood gas samples were analyzed using a standard blood gas analyzer (ABL 725, Radiometer, Copenhagen).

### Statistical Analysis

Data were stored using Microsoft Excel 97 and analyzed using STATA-8 (Stata, College Station TX). The primary analysis was performed using the techniques describe by Bland and Altman [22]. Bias and the limits of agreement were calculated. Bias, or systematic error, is determined by the mean difference between SpO_2 _and SaO_2_, whereas precision, or random error, is determined by the standard deviation of the mean difference. Positive bias means that pulse oximetery overestimates SaO_2 _and negative bias means that it is underestimated. The limits of agreement are the mean difference ± 2SD. Stratified analyses were performed to investigate contributions of lactate, hypoxemia (as estimated by a SaO_2 _<90%), bacteremia, pH, hemoglobin, and the requirement of vasoactive drugs to the relationship of SpO_2 _and SaO_2_. Normally or near-normally distributed variables were reported as means with standard deviations (SD) and non-normally distributed variables as medians with inter-quartile ranges (IQR). Means were compared using the appropriate Student's *t *test. A *P*-value of ≤ 0.05 was considered statistically significant. Given the exploratory nature of the analysis, no correction for multiple analyses was made.

## Results

Ninety patients had simultaneous arterial blood gases and oxygen saturation values recorded. Upon review, 2 results were deemed to be venous samples and were excluded from the analysis. The remaining 88 simultaneous readings were analyzed to determine the bias and limits of agreement. Patient characteristics are reported in Table 1. None of the patients had any recorded history of smoke inhalation or carbon monoxide exposure.

**Table 1:** Summary of patient characteristics.

Variable		Range
Sex (male), n (%)	45 (51%)	NA

Received vasopressors, n (%)	40 (45%)	NA

Positive blood culture in ED, n (%)	29 (33%)	NA

Age (years), mean	57.5	19-89

pH mean (SD)	7.35 (0.15)	6.64-7.65

PaCO_2 _(mmHg), mean (SD)	33.7 (12.04)	12-72

PaO_2 _(mmHg), mean (SD)	106 (77)	43-465

SaO_2 _(%), mean (SD)	91.2 (5.74)	71-98

SpO_2 _(%), mean (SD)	93.9 (4.82)	78-100

Lactate (mmol/L), mean (SD)	3.21 (2.46)	0.6-16

Hemoglobin (g/L), mean (SD)	132 (24)	66-190

Admitting APACHE II score, mean (SD)	20 (10)	3-40

**Figure 1:**
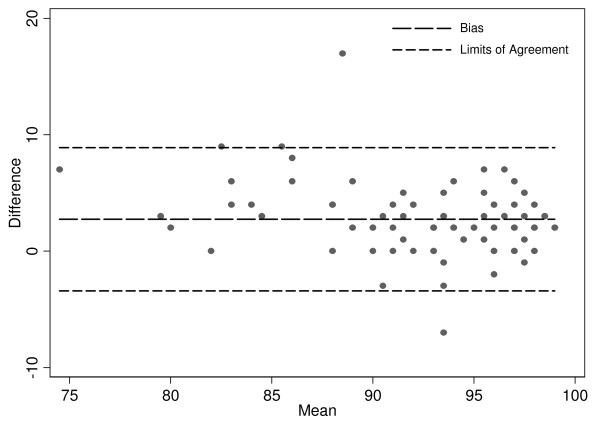
**Bland-Altman plot for bias and limits of agreement**.

The mean ± SD for SpO_2 _was 93.9% ± 4.8% and the mean for SaO_2 _was 90.2% ± 9.7%. Bland Altman analysis indicated a bias of 2.75% and limits of agreement -3.4% and 8.9% (Figure 1). The effects of hypoxemia (SaO_2 _<90), lactate (>2 mmol/L and >4 mmol/L), acidosis (pH < 7.35), anemia (below median and quartile hemoglobins), bacteremia (positive cultures from ED draw), and requirement for vasoactive drugs on bias and limits of agreement are shown in Table 2. The mean differences (SpO_2 _- SaO_2_) in hypoxemic patients was 4.92% and in non-hypoxemic patients was 1.89% (p < 0.004). All 28 patients with SpO_2 _values ≥ 98% had SaO_2 _values > 90%. Of the 31 patients with SpO_2 _values ranging from 94-97%, 3 (9.7%) had SaO_2 _values < 90%. Eleven (50%) of the 22 patients with SpO_2 _values from 90-93% have SaO_2 _values < 90%. Accuracy of SpO_2 _was not demonstrated to be affected by acidoisis, hyperlactatementa, anemia, or vasoactive drug use in this cohort. However, these variables markedly decreased precision (Table 2).

**Table 2:** Pulse oximeter bias (mean SpO_2 _- SaO_2_) (%) in different subgroups of patients.

Parameter	Subgroup	n	Bias (%)	*p*	Limits of Agreement
SaO2	<90	27	4.92	0.004	-2.2 to 12.0

	≥ 90	67	1.89		-2.9 to 6.7

					

Lactate	>2	52	2.58	0.40	-4.2 to 9.3

	≤ 2	35	3.14		-1.9 to 8.1

					

	>4	25	2.52	0.67	-6.2 to 11.2

	≤ 4	62	2.92		-1.8 to 7.8

					

pH	<7.35	43	3.15	0.30	-4.6 to 10.9

	≥ 7.35	51	2.43		-2.1 to 6.9

					

Hemoglobin	<136	46	3.00	0.42	-4.1 to 10.1

	≥ 136	42	2.48		-2.4 to 7.4

					

	<119	22	3.41	0.38	-5.3 to 12.2

	≥ 119	66	2.53		-2.5 to 7.6

					

Blood culture	Positive	29	3.66	0.06	-3.8 to 11.1

	Negative	47	2.21		-3.3 to 7.7

					

Vasopressors	Yes	40	3.05	0.43	-4.8 to 10.9

	No	48	2.50		-1.8 to 6.8

Bias was higher in those who experienced ICU mortality but this did not reach statistical significance (3.9% vs. 2.5%, p = 0.28). There was no significant association between bias and admitting APACHE II score.

## Discussion

There is little data on the accuracy of pulse oximetry in critically ill ED patients. Studies in both the ED and the ICU have produced mixed results and were comprised of mostly small and heterogenous patient populations that did not include sepsis. Data on severely septic patients, a population where tissue hypoxia is particularly prevalent and important [18,19], is lacking.

Studies of pulse oximeter accuracy in populations of critically ill patients have revealed mixed results. Whereas some studies of critically ill patients have found that SpO_2 _has underestimated SaO_2 _[1,5,8,9], others have found the opposite [2,3,7]. Studies of small numbers of heterogenous ICU patients reported biases of -2.5% to 2.5% [1,3,5,7]. In similar studies specifically in patients with severe sepsis and septic shock, results are again conflicting. In a prospective study of 20 general ICU patients, Secker and Spiers [9] reported that pulse oximetry significantly underestimated SaO_2 _by a mean of 1.4% (p < 0.001) in patients with septic shock but this bias was not significantly different relative to those without septic shock. In contrast Ibanez and colleagues [7] reported that ear pulse oximetry underestimated SaO_2 _by a mean difference (± SD) of 2.5% ± 4% (p = 0.009) however accuracy was significantly greater in the 13 shock patients than in the non-shock patients, with mean differences (± SD) of 1.7% ± 5.2% and 3.4% ± 2.8% (p = 0.002), respectively. Although there was less bias in the shock group, pulse oximetry was significantly less precise in this group. These mixed results may be partly explained by the use of different pulse oximeters in each study, as bias has been shown to be oximeter-specific [23].

It has been postulated that sepsis-induced arteriolar dilation and the opening of arteriovenous shunts [9,12] may increase venous pulsatility potentially leading pulse oximeters to identify pulsating venous blood as being arterial [11]. The lower venous oxygen saturation of venous blood would be expected to dilute the arterial fraction resulting in underestimation of SaO_2_. Similar to the work of Ibanez [7], we demonstrated that pulse oximetry overestimated SaO_2 _questioning the proposed mechanism of Secker and Spiers [9]. We measured accuracy earlier in the course of disease while resuscitation was ongoing. It is possible that our patients were incompletely resuscitated at the time of measurement, affecting the proportion of open ateriovenous shunts. Alternatively, bias could be a marker of local heterogeneity of microvascular flow. If microvascular flow disturbance was a marker of severity of illness, bias could offer additional prognostic information. In this study bias was not associated with APACHE II score but was non-significantly higher in non-survivors.

The factors influencing pulse oximeter accuracy have not been well studied. Our data confirm previous reports of the detrimental effect of hypoxemia on bias [1,5,24]. Possible reasons for decreased pulse oximeter accuracy with hypoxemia include lack of reliable human calibration data during extreme hypoxia and an increased proportion of reduced hemoglobin in hypoxic states, which can exacerbate error in the absorption ratio [1,25] The need for vasoactive drugs in the ED did not significantly affect the accuracy of pulse oximetry in our study. As pulse oximters are dependent upon arterial pulsatility, vasopressors may theoretically increase bias via decreased pulsatility secondary to arteriolar vasoconstriction [25]. The few ICU studies that have included vasopressor-dependent patients have revealed mixed results. Bias was significantly increased in a subset of 13 patients receiving vasoactive drugs compared to 89 patients not receiving the drugs, with biases of 0.70 and -0.11 (p < 0.05), respectively [1]. In another study of 18 ICU patients [6], signal failure occurred in 2 of 9 patients receiving vasoactive drugs.

Our study has limitations that warrant discussion. As we studied a relatively homogenous patient population, our results should not be generalized to non-septic critically ill patients or to those outside the initial ED phase of severe sepsis. The retrospective nature of the trial precluded any reliable assessment of the validity of the pulse oximeter waveform as the SpO_2 _was recorded. Moreover, as was the case in other studies of pulse oximeter accuracy [1,7], we did not include a control group, complicating the proportion of bias that can be attributed to severe sepsis. Additionally, we could only report if vasopressors were given in the ED and not specifically if they were given at the time that the specific ABG was drawn. We did not control for all factors that may influence bias. For example, we did not account for other physiologic variables, such as inspiratory pressure [26] or PaCO_2 _that may affect bias. Nail polish may also affect SpO2 readings [27]. The standard of care at our institution is to place the pulse oximeter probe on a digit without nail polish or if all digits have nail polish to remove it with nail polish remover. As this is not routinely charted, our retrospective study could not audit this practice. Finally, despite being the largest study of pulse oximetry accuracy in sepsis, our sample size may have been insufficient, particularly so in the subset analyses.

## Conclusion

In conclusion, in a group of ED patients with severe sepsis or septic shock, pulse oximters overestimated measured SaO_2 _by a mean of 2.75%. Hypoxemia significantly contributed to pulse oximeter bias whereas acidosis, hyperlactatemia, decreased Hb level, bacteremia, and the need for vasopressors did not. Clinicians should be aware of the bias and the wide limits of agreement when considering SpO_2 _readings in the management of patients with severe sepsis and septic shock especially when values are <98%. When SaO_2 _needs to be determined with a high degree of accuracy in such patients arterial blood gases are recommended.

## Abbreviations

ABG: arterial blood gas; ED: emergency department; FiO_2_: fraction of inspired oxygen; ICNARC: Intensvice Care National Audit & Research Centre; ICU: intensive care unit; SaO_2_: arterial hemoglobin saturation; SpO_2_: pulse oximeter oxygen saturation.

## Competing interests

The authors declare that they have no competing interests.

## Authors' contributions

BW conceived of the design of the study, carried out the chart review, and drafted the manuscript. HC assisted with the chart review. JL and DZu participated in the drafting of the manuscript. DZy participated in the design of the study, performed the statistical analysis, and helped draft the manuscript. All authors read and approved the final manuscript.

## Pre-publication history

The pre-publication history for this paper can be accessed here:

http://www.biomedcentral.com/1471-227X/10/9/prepub

## References

[B1] Louw A Van de Cracco C Cerf C Harf A Duvaldestin P Lemaire F Brochard L Accuracy of pulse oximetry in the intensive care unit Intensive Care Med 2001 27 10 1606 1613 10.1007/s001340101064 11685301

[B2] Lee WW Mayberry K Crapo R Jensen RL The accuracy of pulse oximetry in the emergency department Am J Emerg Med 2000 18 4 427 431 10.1053/ajem.2000.7330 10919532

[B3] Seguin P Le Rouzo A Tanguy M Guillou YM Feuillu A Malledant Y Evidence for the need of bedside accuracy of pulse oximetry in an intensive care unit Crit Care Med 2000 28 3 703 706 10.1097/00003246-200003000-00017 10752818

[B4] Jubran A Pulse oximetry Intensive Care Med 2004 30 11 2017 2020 10.1007/s00134-004-2399-x 15278272

[B5] Jubran A Tobin MJ Reliability of pulse oximetry in titrating supplemental oxygen therapy in ventilator-dependent patients Chest 1990 97 6 1420 1425 10.1378/chest.97.6.1420 2347228

[B6] Mihm FG Halperin BD Noninvasive detection of profound arterial desaturations using a pulse oximetry device Anesthesiology 1985 62 1 85 87 10.1097/00000542-198501000-00020 3966675

[B7] Ibanez J Velasco J Raurich JM The accuracy of the Biox 3700 pulse oximeter in patients receiving vasoactive therapy Intensive Care Med 1991 17 8 484 486 10.1007/BF01690773 1797894

[B8] Perkins GD McAuley DF Giles S Routledge H Gao F Do changes in pulse oximeter oxygen saturation predict equivalent changes in arterial oxygen saturation? Crit Care 2003 7 4 R67 10.1186/cc2339 12930558 PMC270702

[B9] Secker C Spiers P Accuracy of pulse oximetry in patients with low systemic vascular resistance Anaesthesia 1997 52 2 127 130 10.1111/j.1365-2044.1997.32-az0062.x 9059094

[B10] Severinghaus JW Koh SO Effect of anemia on pulse oximeter accuracy at low saturation J Clin Monit 1990 6 2 85 88 10.1007/BF02828282 2352007

[B11] Ralston AC Webb RK Runciman WB Potential errors in pulse oximetry. I. Pulse oximeter evaluation Anaesthesia 1991 46 3 202 206 10.1111/j.1365-2044.1991.tb09410.x 2014898

[B12] Spronk PE Zandstra DF Ince C Bench-to-bedside review: sepsis is a disease of the microcirculation[see comment] Crit Care 2004 8 6 462 468 10.1186/cc2894 15566617 PMC1065042

[B13] Broome IJ Mills GH Spiers P Reilly CS An evaluation of the effect of vasodilatation on oxygen saturations measured by pulse oximetry and venous blood gas analysis Anaesthesia 1993 48 5 415 416 10.1111/j.1365-2044.1993.tb07017.x 8317652

[B14] Hummler HD Engelmann A Pohlandt F Hogel J Franz AR Accuracy of pulse oximetry readings in an animal model of low perfusion caused by emerging pneumonia and sepsis Intensive Care Med 2004 30 4 709 713 10.1007/s00134-003-2116-1 14722632

[B15] Hummler HD Pohlandt F Franz AR Pulse oximetry during low perfusion caused by emerging pneumonia and sepsis in rabbits Crit Care Med 2002 30 11 2501 2508 10.1097/00003246-200211000-00016 12441761

[B16] Shoemaker WC Appel PL Kram HB Bishop MH Abraham E Temporal hemodynamic and oxygen transport patterns in medical patients. Septic shock Chest 1993 104 5 1529 1536 10.1378/chest.104.5.1529 8222819

[B17] Hotchkiss RS Karl IE The pathophysiology and treatment of sepsis[see comment] N Engl J Med 2003 348 2 138 150 10.1056/NEJMra021333 12519925

[B18] Sakr Y Dubois MJ De Backer D Creteur J Vincent JL Persistent microcirculatory alterations are associated with organ failure and death in patients with septic shock Crit Care Med 2004 32 9 1825 1831 10.1097/01.CCM.0000138558.16257.3F 15343008

[B19] Ince C The microcirculation is the motor of sepsis Crit Care 2005 9 Suppl 4 S13 9 10.1186/cc3753 16168069 PMC3226164

[B20] Bone RC Balk RA Cerra FB Dellinger RP Fein AM Knaus WA Schein RM Sibbald WJ Definitions for sepsis and organ failure and guidelines for the use of innovative therapies in sepsis. The ACCP/SCCM Consensus Conference Committee. American College of Chest Physicians/Society of Critical Care Medicine[see comment] Chest 1992 101 6 1644 1655 10.1378/chest.101.6.1644 1303622

[B21] Ferreira FL Bota DP Bross A Melot C Vincent JL Serial evaluation of the SOFA score to predict outcome in critically ill patients JAMA 2001 286 14 1754 1758 10.1001/jama.286.14.1754 11594901

[B22] Bland JM Altman DG Statistical methods for assessing agreement between two methods of clinical measurement Lancet 1986 1 8476 307 310 2868172

[B23] Severinghaus JW Naife KH Koh SO Errors in 14 pulse oximeters during profound hypoxia J Clin Monit 1989 5 2 72 81 10.1007/BF01617877 2723709

[B24] Thrush D Hodges MR Accuracy of pulse oximetry during hypoxemia South Med J 1994 87 4 518 521 10.1097/00007611-199404000-00019 8153783

[B25] Szaflarski NL Cohen NH Use of pulse oximetry in critically ill adults Heart Lung 1989 18 5 444 453 2777564

[B26] Scheller J Loeb R Respiratory artifact during pulse oximetry in critically ill patients Anesthesiology 1988 69 4 602 603 10.1097/00000542-198810000-00025 3177921

[B27] Hinkelbein J Genzuerker HV Sogl R Fiedler F Effects of nail polish on oxygen saturation determined by pulse oximetry in critically ill patients Resuscitation 2007 72 1 82 91 10.1016/j.resuscitation.2006.06.024 17098347

